# Spatio‐Temporal Variations in Carbon Isotope Discrimination Predicted by the JULES Land Surface Model

**DOI:** 10.1029/2022JG007041

**Published:** 2022-12-08

**Authors:** Lewis Palmer, Iain Robertson, Aliénor Lavergne, Deborah Hemming, Neil J. Loader, Giles Young, Darren Davies, Katja Rinne‐Garmston, Sietse Los, Jamie Williams

**Affiliations:** ^1^ Department of Geography Swansea University Swansea UK; ^2^ Modelling and Informatics Soils, Crops, and Water RSK ADAS Limited Bristol UK; ^3^ Department of Geography and Environmental Science University of Reading Reading UK; ^4^ Department of Physics Imperial College London London UK; ^5^ Met Office Exeter UK; ^6^ Natural Resources Institute Finland (Luke) Helsinki Finland; ^7^ Wetland Conservation Unit Wildfowl and Wetland Trust (WWT) Gloucestershire UK

**Keywords:** carbon isotope discrimination, tree‐ring, JULES, land‐surface model, UK

## Abstract

Stable carbon isotopes in plants can help evaluate and improve the representation of carbon and water cycles in land‐surface models, increasing confidence in projections of vegetation response to climate change. Here, we evaluated the predictive skills of the Joint UK Land Environmental Simulator (JULES) to capture spatio‐temporal variations in carbon isotope discrimination (Δ^13^C) reconstructed by tree rings at 12 sites in the United Kingdom over the period 1979–2016. Modeled and measured Δ^13^C time series were compared at each site and their relationships with local climate investigated. Modeled Δ^13^C time series were significantly correlated (*p* < 0.05) with tree‐ring Δ^13^C at eight sites, but JULES underestimated mean Δ^13^C values at all sites, by up to 2.6‰. Differences in mean Δ^13^C may result from post‐photosynthetic isotopic fractionations that were not considered in JULES. Inter‐annual variability in Δ^13^C was also underestimated by JULES at all sites. While modeled Δ^13^C typically increased over time across the UK, tree‐ring Δ^13^C values increased only at five sites located in the northern regions but decreased at the southern‐most sites. Considering all sites together, JULES captured the overall influence of environmental drivers on Δ^13^C but failed to capture the direction of change in Δ^13^C caused by air temperature, atmospheric CO_2_ and vapor pressure deficit at some sites. Results indicate that the representation of carbon‐water coupling in JULES could be improved to reproduce both the trend and magnitude of interannual variability in isotopic records, the influence of local climate on Δ^13^C, and to reduce uncertainties in predicting vegetation‐environment interactions.

## Key Points

- Joint UK Land Environmental Simulator (JULES) predicted well the tree‐ring Δ^13^C of oaks in eight out of 12 UK sites but underestimated inter‐annual variations and mean values
- JULES captured the environmental drivers of Δ^13^C, but the model failed to reproduce the direction of change in Δ^13^C at some individual sites
- More tree‐ring chronologies are needed to improve understanding of spatio‐temporal changes in Δ^13^C for UK broadleaf deciduous trees

## 1 Introduction

Temperate broadleaf deciduous woodlands are important carbon sinks (Thomas et al., 2011), contributing to nearly 60% of the total worldwide forest carbon uptake (Pan et al., 2011), helping to offset anthropogenic greenhouse gas emissions and thus mitigating climate change. At present, broadleaf deciduous forests are heavily fragmented in central Europe (Haddad et al., 2015), due to large‐scale deforestation over the past centuries. The United Kingdom (UK), in particular, has replaced these forests with more commercially favorable conifers, affecting biodiversity and potentially impacting the climate mitigation potential of forests through changes in albedo and evapotranspiration (Naudts et al., 2016). The Paris Agreement (2015) clearly states that improved land use and forest management are required to help reduce atmospheric CO_2_ emissions in 2030 by up to 55% and reach Net‐Zero emissions by 2050. One strategy is to create new woodlands, the extent of which could reach around 2 Mha of land in UK (Bradfer‐Lawrence et al., 2021). In a scenario consistent with the Net Zero pathway, the new woodlands should be dominated by broadleaf trees with different percentages for northern and southern of UK (67% in Wales and Northern Ireland, 80% in England but 50% in Scotland; Bradfer‐Lawrence et al., 2021). There is still, however, a need to investigate how temperate broadleaf deciduous forests in UK are impacted by environmental changes and how well their responses to climate are predicted in land‐surface models (LSMs) to increase confidence in the projections of this biome's potential to mitigate climate change. Evaluation of LSMs will also improve understanding of ecosystem functions in response to future climate scenarios.

Tree rings are powerful proxies for understanding plant ecophysiological responses to climate change because of their high temporal (intra‐ and inter‐annual) resolution and their widespread spatial distribution (McCarroll & Loader, 2004). Parameters such as ring‐widths and stable (oxygen and carbon) isotopic measurements have been widely used in dendroecology (Belmecheri & Lavergne, 2020; de Boer et al., 2019; Duarte et al., 2017; ) and dendroclimatology (Büntgen et al., 2021; Helama et al., 2018; Loader et al., 2020; G. H. F. Young et al., 2019). The carbon isotopic composition of leaves, that is, the ratio of ^13^C–^12^C compared to that of a reference material expressed using the delta notation δ^13^C (Coplen, 1995), varies between C_3_ plant species and is strongly influenced by photosynthetic and stomatal processes (Farquhar et al., 1982; Lloyd & Farquhar, 1994). Trees assimilate the lighter ^12^C more readily than ^13^C in a process termed isotopic discrimination (Δ^13^C) (Park & Epstein, 1960). Discrimination occurs during CO_2_ diffusion from the atmosphere through the stomata, mesophyll and subsequently during CO_2_ carboxylation by the enzyme Rubisco in the chloroplast (Farquhar et al., 1982). The environmental variables that influence Δ^13^C on interannual timescales include the concentration and isotopic composition of atmospheric CO_2_, solar radiation, air temperature, vapor pressure deficit, atmospheric pressure via elevation (Cornwell et al., 2018; Hafner et al., 2014; Körner et al., 1991; Wang et al., 2017; G. H. Young et al., 2010) as well as plant‐available water via changes in precipitation and soil moisture (Diefendorf et al., 2010; Kohn, 2010). Pollution and nutrient variability also influence Δ^13^C (Cernusak et al., 2013; Domingues et al., 2010; Field, 1986; Linzon, 1972; Martin et al., 1988; Rinne et al., 2010; Savard et al., 2004). Since the supply and demand for CO_2_ by plants to grow and maintain essential physiological processes are the dominant controls of plant isotopic discrimination (Raczka et al., 2016), Δ^13^C records derived from tree‐ring δ^13^C can provide insights into plant physiological adjustments to different climatic conditions (Lavergne et al., 2022). Consequently, the capacity of LSMs to predict the coupled carbon and water cycles can be tested by using tree‐ring derived Δ^13^C (Belmecheri & Lavergne, 2020; Keller et al., 2017). Improved modeling of stomatal functions in LSMs would lead to more reliable estimates of the impact of changes in stomatal conductance on climate as they can modulate the latent heat fluxes (Bodin et al., 2013). Current LSMs predict relatively well modern δ^13^C chronologies, however, when timescales are increased to greater than 20 years, the model predictive skills tend to be reduced (Barichivich et al., 2021). There is therefore a need to develop datasets with long temporal resolution that can be used for evaluating LSMs.

Stable carbon isotopes have been implemented into several LSMs. Raczka et al. (2016) predicted Δ^13^C values using CLM4.5 model and found that it overestimated Δ^13^C values compared to those derived from tree rings in Colorado (USA). However, by using revised stomatal model parameters, the model predictive skills were increased significantly. Similarly, Keller et al. (2017) investigated the ability of both CLM4.5 and LPX‐Bern models to reproduce Δ^13^C and intrinsic water use efficiency (iWUE) trends over the twentieth century from a world‐wide tree‐ring δ^13^C data set. They found that the predictive skills of CLM4.5 were lower than those from LPX‐Bern primarily because of issues with the implementation of stomatal conductance and assimilation in CLM4.5. Elsewhere, Churakova et al. (2015) compared simulations from the ORCHIDEE model with tree‐ring δ^13^C time series of Siberian larch and found that the inter‐annual variability in δ^13^C was underestimated by the model; the model captured up to 26% of the measured variability in δ^13^C. They also found offsets of approximately 4‰ between modeled and tree‐ring derived δ^13^C. In a more recent study by Barichivich et al. (2021), however, the authors found that ORCHIDEE was able to predict the inter‐annual variability in tree‐ring carbon isotopes more accurately, capturing 30%–46% of the variance in the observations. They also revealed similar performances by LPX‐Bern and MAIDENiso models. Bodin et al. (2013) evaluated three stomatal models (i.e., Ball‐Berry (Ball et al., 1987), COX (Cox et al., 1998), and SPA (Williams et al., 1996)) within Joint UK Land Environment Simulator (JULES with measured δ^13^C values derived from trees in Northern Europe. They found that all three models performed poorly in reproducing the measured inter‐annual variability of δ^13^C but that the SPA stomatal model was the most effective. Similarly, Lavergne et al. (2022) tested four different stomatal models in JULES to predict leaf‐intercellular CO_2_ and therefore Δ^13^C (i.e., Leuning, 1995; Jacobs, 1994; Medlyn et al., 2011; Prentice et al., 2014) and showed that the bias between measured and predicted Δ^13^C was reduced when using the Prentice et al. (2014) model, and the predictions of canopy‐level carbon and water fluxes were also improved. These studies demonstrate the importance of evaluating prediction of LSMs using stable carbon isotopes.

Here, we explore spatio‐temporal variations of Δ^13^C in broadleaf deciduous oak trees growing at 12 sites across UK and compare them with those predicted by JULES over the period 1979–2016. The study focuses upon oaks growing in temperate broadleaf deciduous woodlands in the UK as these forests have the potential to sequester significant amounts of carbon (Thomas et al., 2011) and are important to meet the Net Zero pathway (Bradfer‐Lawrence et al., 2021). We then investigate the environmental dependencies of Δ^13^C measured in the tree‐ring network and predicted by JULES. We address the following questions: (a) How effective is JULES at predicting Δ^13^C of UK broadleaf deciduous trees? (b) Can JULES capture the response of Δ^13^C to local climate conditions? And finally, (c) What are the main drivers of Δ^13^C variations across the UK?

## 2 Materials and Methods

### 2.1 Description of JULES Model

JULES is the land‐surface component of the UK Earth System Model (Sellar et al., 2019) simulating the fluxes of carbon, water and energy between the atmosphere and the land surface. JULES can be used independently or coupled to the Met Office Unified Model (Cullen, 1993) and thus can impact weather forecasting and climate change projections. The modular structure of JULES allows for the interaction of different land‐surface processes (e.g., carbon cycle, dynamic vegetation, hydrological cycle). Consequently, JULES can be used to assess the impact of a single process on the entire ecosystem (Best et al., 2011). JULES represents the vegetation in nine plant functional types (Harper et al., 2016), including broadleaf deciduous trees. The model requires a series of meteorological forcing variables such as downward shortwave and longwave radiation, wind speed, precipitation (including rainfall and snowfall), air humidity, surface pressure and air temperature.

In this study, we used JULES simulations produced recently by Lavergne et al. (2022) based on JULES vn5.6 (see Harper et al. (2018) for parameter values) with a new carbon isotopic capability. This model configuration enables the calculation of Δ^13^C and therefore δ^13^C in tree‐ring cellulose. The model was driven by WFDEI‐WATCH data set which has a spatial resolution of 0.5° × 0.5° and daily temporal resolution spanning the period 1979–2016 (see Weedon et al. (2014) for a full description), following Lavergne et al. (2022) who ran the model at the global scale. Atmospheric CO_2_ concentration data from NOAA/ESRL Global Monitoring Laboratory, Boulder Colorado, USA (https://gml.noaa.gov/ccgg/trends/) and δ^13^CO_2_ data from Graven et al. (2017) were also used to run the model. We used a fixed land cover mask based on the European Space Agency's Land Cover Climate Change Initiative global vegetation distribution. A more detailed description of the model version and configuration can be found in Lavergne et al. (2022). Δ^13^C was estimated including both photorespiratory and mesophyll effects as:

(1) $$\Delta^{13}C=a\frac{c_{a}-c_{i}}{c_{a}}+b\frac{c_{c}}{c_{a}}-f\frac{\Gamma_{\;c}^{*}}{c_{a}}+a_{m}\frac{c_{i}-c_{c}}{c_{a}}\;$$

where *a* (4.4‰), *a*_*m*_ (1.8‰), *b* (28 ± 2‰; Ubierna & Farquhar, 2014), *f* (12 ± 4‰) are the isotopic fractionation effects due to diffusion of CO_2_ through the stomata and the mesophyll, RuBisCO carboxylation, photorespiration, respectively. Γ* is the photorespiratory compensation point (Pa). *c*_a_, *c*_i_, and *c*_c_ are the ambient, leaf‐intercellular and chloroplastic partial pressure of CO_2_ (Pa). *c*_i_ was calculated using the Prentice et al. (2014) stomatal model as:

(2a) $$c_{i}=\left(c_{a}-\Gamma^{*}\right)\frac{\xi }{\xi +\sqrt{D}}+\Gamma^{*}$$

(2b) $$\xi =\;\sqrt{\beta \frac{\left(K+\Gamma^{*}\right)}{1.6_{\eta *}}}$$

where β represents the cost factors of transpiration and carboxylation at 25°C, which may vary with plant‐available soil water (Lavergne et al., 2020) but is assumed constant here because mechanistic soil water stress formulations to incorporate into JULES are yet to be proposed. We used default maximum rate of carboxylation values for deciduous broadleaf forests of 57.25 at 25°C (following Harper et al., 2016). *K* is the Michaelis‐Menten constant for Rubisco‐limited photosynthesis (Pa) and η* is the viscosity of water (unitless), which depends on air temperature and atmospheric pressure, but has been assumed constant (equal to one) for these simulations (see Lavergne et al., 2022). *c*_c_ was estimated as in Wang et al. (2017):

(3a) $$c_{c}=\left(c_{a}-\Gamma_{c}^{*}\right)\frac{\xi_{c}}{\xi_{c}+\;\sqrt{D}}+\Gamma_{c}^{*}$$

(3b) $$\xi_{c}=\;\sqrt{\beta_{c}\frac{\left(K_{c}+\;\Gamma_{c}^{*}\right)}{1.6_{\eta *\;}\left(1+\;g_{sc}/g_{m}\right)}}$$

where *β*_c_, Γ_c_*, and *K*_c_ are equivalent to *β*, Γ*, and *K* when *g*_m_ (mesophyll conductance) is assumed to be finite. *g*_sc_ refers to the leaf‐level stomatal conductance (mol m^−2^ s^−1^). This equation assumes a constant ratio of *g*_sc_ to *g*_m_ (Lavergne et al., 2022).

### 2.2 Tree‐Ring δ^13^C Data

**Figure 1:**
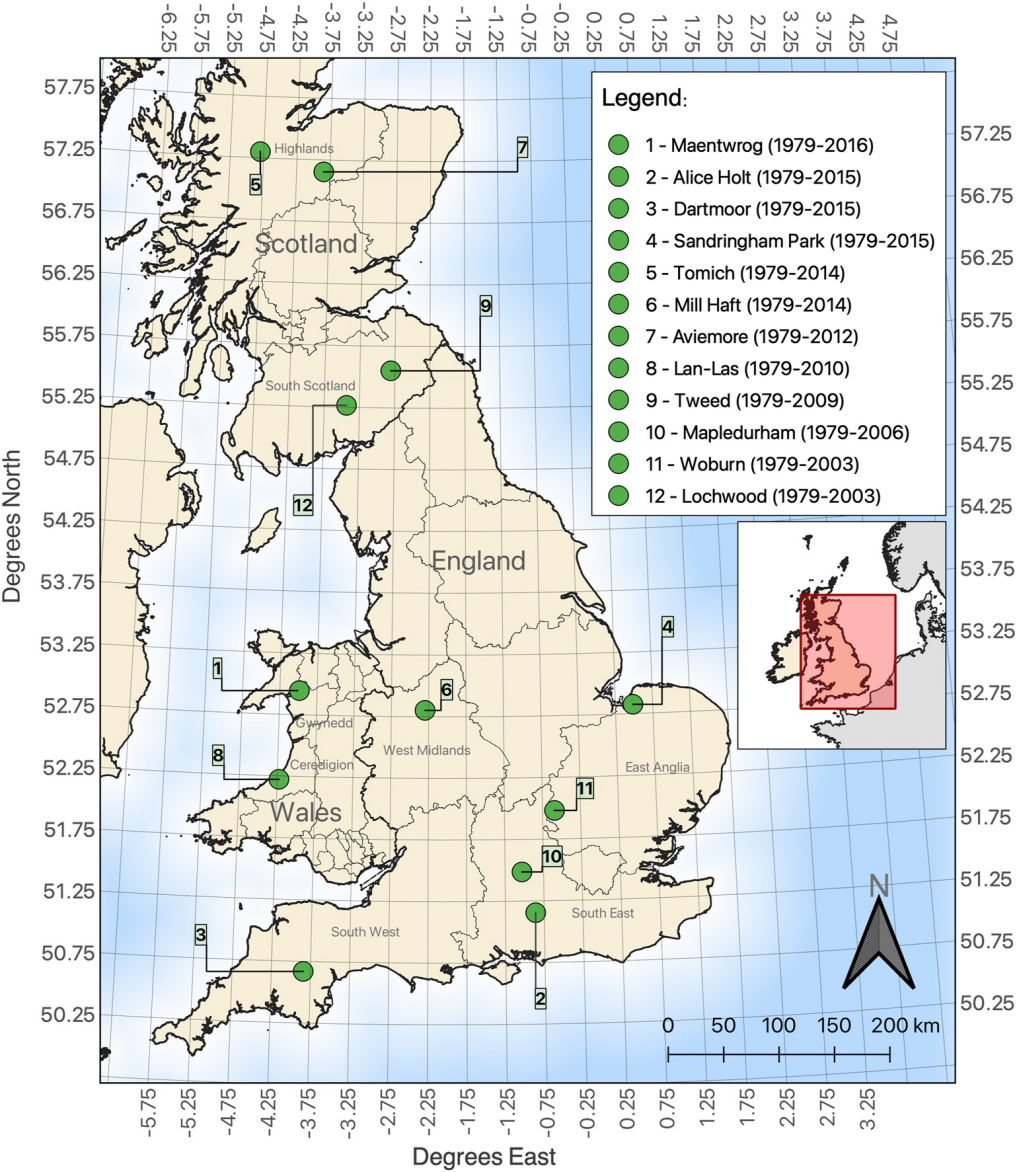
Geographical locations of the study sites with a 0.5° × 0.5° grid.

We used absolutely dated (Loader et al., 2008; Stokes & Smiley, 1968) existing tree‐ring δ^13^C time series from 12 datasets sampled across the UK. All 12 chronologies correspond to broadleaf deciduous trees, more specifically pedunculate oak (*Quercus robur* L.) and sessile oak (*Quercus petraea* (Matt.) Liebl.). Carbon isotopes were determined on latewood cellulose. Even though some δ^13^C series started before 1979, we only considered the 1979–2016 period for the model‐data comparison. Given the limited UK‐based δ^13^C datasets, we also considered time series that ended before 2016 (Table 1). The study sites were well distributed across the UK (see Figure 1 for geographical location of study sites). The δ^13^C chronologies comprised two sites in Wales, four in Scotland, with the remaining six in central and southern England. All stable carbon isotope time series analyzed here span at least 25 years within the period 1979 to 2016 (Table 1).

**Table 1:** Measured Tree‐Ring δ^13^C Chronologies Used in This Study, With Reference to the Original Source or Author(s)

Site	Source	Published/Unpublished	Length (years)	Lat/Lon	Elevation (meters)	Dominant tree species
Maentwrog	Loader and Sladden	Unpublished	38	52.95, −3.99	27–55	*Q. petraea*
Alice Holt	Loader and Young	Unpublished	37	51.18, −0.85	107	*Q. robur*
Dartmoor	Loader and Jones	Unpublished	37	50.67, −3.84	217	*Q. petraea*
Sandringham Park	Robertson et al.	Unpublished	37	52.83, 0.50	38	*Q. robur*
Tomich	Loader and Rowe	Unpublished	36	57.30, −4.80	184	*Q. petraea*
Mill Haft	Loader et al.	Unpublished	36	52.80, −2.30	108	*Q. robur*
Aviemore	McCarroll et al. (2017)	Published	34	57.15, −3.84	300	*Q. robur*
Lan‐las	Young et al. (2015)	Published	32	52.22, −4.22	111	*Q. petraea*
Tweed	Williams	Unpublished	31	55.55, −2.80	190	*Q. robur*
Mapledurham	Young et al. (2015)	Published	28	51.50, −1.00	70	*Q. robur/Q. petraea*
Woburn	Rinne et al. (2013)	Published	25	51.98, −0.58	150	*Q. robur*
Lochwood	Loader et al. (2008)	Published	25	55.27, −3.43	175	*Q. robur*

*Note.* Coordinates are given in the form of decimal latitude/longitude.

Since the onset of the global Industrial Revolution (c. 1850), the release of isotopically light carbon dioxide (depleted in ^13^C) into the atmosphere primarily from anthropogenic burning of fossil fuels has resulted in a strong reduction in the δ^13^CO_2_ of approximately 1.93‰ since 1850 (Belmecheri & Lavergne, 2020) in a process known as the Suess effect (Keeling, 1979). Some of the δ^13^C chronologies we used had been mathematically corrected for the Suess effect by the original author(s) (after McCarroll & Loader, 2004). To ensure consistency, the Suess correction therefore had to be removed from these data before calculating Δ^13^C using the following formula:

(4) $$\delta^{13}C_{\text{TR}}=\delta^{13}C_{\text{TR}\left(\text{corrected}\right)}+\left(\delta^{13}{\text{CO}}_{2}-\delta^{13}{\text{CO}}_{2\text{PI}}\right)$$

This rearranged equation is based on a Suess correction equation from Belmecheri and Lavergne (2020), where δ^13^CO_2PI_ is the δ^13^CO_2_ value prior to industrialization equal to −6.61‰ (year 1850). Subsequently, tree‐ring derived Δ^13^C (noted Δ^13^C_TR_) was calculated from the de‐corrected δ^13^C_TR_ data as:

(5) $$\Delta^{13}C_{\text{TR}}=\frac{\delta^{13}{\text{CO}}_{2}-\left(\delta^{13}C_{\text{TR}}-d\right)}{1+\left(\delta^{13}C_{\text{TR}}-d\right)/1000}$$

where *d* is the total post‐photosynthetic isotopic fractionations that occurred before the carbohydrates synthetized in the leaf are incorporated into the wood. There is still a debate in the literature around the true value of *d,* but it is believed to be approximately 2.1‰ between the leaf material and α‐cellulose (Belmecheri & Lavergne, 2020; Frank et al., 2015). We used this value in our calculation.

### 2.3 Comparison and Analyses

We used the software R (R Core Team, 2020) to analyze the data and model simulations. It is widely acknowledged that across the UK, oak latewood is predominantly formed in the summer months and as such, the main climatic influence upon carbon isotopes in oak latewood may be approximated, for the purposes of this study, as that of July and August (Aykroyd et al., 2001; McCarroll & Loader, 2004; Robertson et al., 1997; G. H. F. Young et al., 2012). Average July‐August Δ^13^C values (hereafter Δ^13^C_predicted_) were therefore extracted for each site from the 0.5° × 0.5° spatial resolution grid points of the “broadleaf deciduous forest” layer. Simulated Δ^13^C_predicted_ values were compared with tree‐ring inferred Δ^13^C_TR_ in terms of their absolute mean values, trends, and inter‐annual variabilities.

The Spearman's rank correlation coefficient was calculated between modeled and measured Δ^13^C chronologies to determine the relationship between both time series. A strong correlation between measured and modeled Δ^13^C (rho > 0.5) with a *p*‐value of <0.001 indicates high model predictive skills, although weaker correlations (rho < 0.5) can also indicate effective and realistic modeling of Δ^13^C if *p*‐value is less than 0.05.

Inter‐annual variability in both modeled and measured Δ^13^C was expressed as the standard deviation from the mean:

(6) $$\sigma =\sqrt{\frac{\Sigma {\left(x_{i}-\mu \right)}^{2}}{N}}$$

where *x*_*i*_ refers to each value from the population, *μ* is the population mean, and *N* is the population size.

To test whether there was a significant difference in the variability of both datasets, we used the non‐parametric Wilcoxon's matched‐pairs signed‐ranks test. We then investigated the environmental drivers of measured and modeled Δ^13^C using the WFDEI‐WATCH climate input data used to run JULES (Weedon et al., 2014), which extends between 1979 and 2016. These data were averaged over July and August to reproduce the latewood climate signal present in the tree‐ring data. We ran multiple linear regression models for both Δ^13^C_TR_ and Δ^13^C_predicted_ against environmental drivers to determine how much of the variability in measured and modeled Δ^13^C were caused by local climate. Since atmospheric CO_2_ concentration ([CO_2_]_atm_), air temperature (*T*_air_), vapor pressure deficit (VPD), and atmospheric pressure via elevation (*z*) are the main drivers of *c*_i_ and thus Δ^13^C at interannual timescales (Cernusak et al., 2013; Cornwell et al., 2018; Diao et al., 2020; Körner et al., 1991; Wang et al., 2017), we considered these variables in the regression models. Both photosynthetically active radiation (PAR) and plant‐available soil water can also impact Δ^13^C (Cernusak et al., 2013; Diefendorf et al., 2010; Hafner et al., 2014; Kohn, 2010; G. H. Young et al., 2010) but were not directly accounted for in the JULES model Δ^13^C predictions and thus excluded from the regression models.

## 3 Results

We found an offset between measured and predicted Δ^13^C values in several sites (Table 2). The largest offsets were at Woburn ranging from 0.81‰ in 1985 to 2.55‰ in 2002. In contrast, Sandringham, Mill Haft, and Lochwood exhibited the lowest offsets of 0.00‰. The average offset between Δ^13^C_TR_ and Δ^13^C_predicted_ across all sites was 0.86‰ with a standard deviation of 0.55‰ (Table 2).

**Table 2:** Offsets (in ‰) Between Δ^13^C_TR_ and Δ^13^C_predicted_ at Each Study Site

Year	Maent‐wrog	Alice Holt	Dart‐moor	Sand‐ringham Park	Tomich	Mill Haft	Avie‐more	Lan‐las	Tweed	Maple‐durham	Woburn	Loch‐wood
1979	0.09	0.40	0.22	0.60	0.69	0.38	1.75	0.17	0.68	1.21	2.52	0.18
1980	0.17	1.02	0.72	0.43	0.38	0.68	0.65	0.13	0.74	0.92	2.01	0.50
1981	0.28	0.69	1.14	0.40	0.99	0.18	0.13	0.23	0.06	0.58	2.44	0.47
1982	0.18	0.16	0.85	0.35	1.22	0.42	0.07	0.03	0.66	0.97	1.92	0.33
1983	0.49	0.75	1.42	0.53	1.14	0.70	0.28	0.95	0.21	1.39	2.33	0.86
1984	0.13	0.72	1.44	0.90	1.01	0.66	0.47	1.53	0.21	1.62	1.69	0.99
1985	0.44	0.17	1.05	0.30	1.33	0.52	0.33	0.72	0.69	1.02	0.81	1.05
1986	0.39	0.20	0.79	0.18	0.19	0.28	1.59	0.19	1.40	1.27	1.42	0.78
1987	0.01	0.46	0.73	0.43	1.30	0.99	0.68	0.54	0.99	1.01	0.91	0.72
1988	0.82	0.42	0.98	0.42	1.31	0.00	1.05	0.48	1.42	1.14	1.47	0.25
1989	1.50	0.66	0.93	0.00	0.70	0.68	0.47	0.96	0.36	1.66	1.70	0.16
1990	1.23	0.85	0.74	0.29	1.05	0.57	0.40	1.47	0.45	1.16	2.31	0.26
1991	0.76	0.96	0.52	0.51	0.89	0.74	0.28	1.07	0.64	1.74	2.24	0.36
1992	1.07	1.37	0.84	0.34	1.24	0.56	0.03	0.25	0.50	1.85	1.37	0.43
1993	1.03	0.65	0.93	0.16	0.93	1.06	0.69	0.15	1.15	1.10	1.45	0.22
1994	1.00	1.51	0.95	0.40	0.61	1.09	0.60	0.78	0.60	1.71	2.17	1.42
1995	0.82	1.21	0.94	0.47	1.46	1.12	1.37	1.47	0.22	0.57	1.88	0.54
1996	0.44	1.32	0.42	0.95	1.19	0.86	0.35	0.76	0.76	1.27	1.97	0.32
1997	0.14	1.30	1.50	0.69	1.05	1.34	1.24	0.88	1.09	1.89	2.20	0.44
1998	1.08	1.10	0.65	0.46	1.11	0.70	0.17	0.06	1.67	0.88	1.34	0.00
1999	0.26	1.16	1.24	0.12	0.78	0.67	0.57	0.65	1.25	1.57	1.77	0.15
2000	0.20	1.09	0.99	0.20	1.05	0.94	1.09	0.55	0.95	1.16	2.09	0.18
2001	0.20	1.80	1.23	0.44	0.70	1.25	0.57	0.27	0.80	1.64	2.07	0.50
2002	0.25	1.48	1.95	0.18	1.17	1.66	0.31	0.50	0.61	1.64	2.55	0.42
2003	0.75	1.01	1.26	0.50	0.59	0.81	1.06	0.92	0.67	1.58	1.81	0.82
2004	0.47	1.35	1.24	0.06	0.93	1.26	0.38	0.60	0.94	1.86		
2005	0.43	1.55	1.40	0.82	0.13	0.76	0.68	0.22	0.92	1.80		
2006	0.20	1.58	1.35	0.05	0.94	0.94	1.09	0.94	0.43	1.68		
2007	0.20	1.13	1.26	0.20	1.06	1.16	0.80	0.01	1.03			
2008	0.39	1.37	1.42	0.05	1.08	0.91	1.34	0.02	1.02			
2009	1.36	1.68	1.53	0.04	1.14	2.09	1.93	0.98	0.28			
2010	0.27	1.30	1.04	0.03	0.95	1.23	1.11	0.11				
2011	0.24	1.16	1.30	0.46	0.76	1.00	1.65					
2012	0.86	1.64	1.52	0.43	0.14	1.29	1.50					
2013	0.24	1.80	1.35	0.53	0.10	0.93						
2014	0.52	1.89	1.15	0.27	0.60	0.55						
2015	0.33	1.52	1.69	0.15								
2016	0.36											
Max	1.50	1.89	1.95	0.95	1.46	2.09	1.93	1.53	1.67	1.89	2.55	1.42
Min	0.01	0.16	0.22	0.00	0.10	0.00	0.03	0.01	0.06	0.57	0.81	0.00
Mean	0.52	1.09	1.10	0.36	0.89	0.86	0.79	0.58	0.75	1.35	1.86	0.49
SD	0.39	0.48	0.37	0.24	0.36	0.41	0.53	0.45	0.39	0.38	0.47	0.34

**Figure 2:**
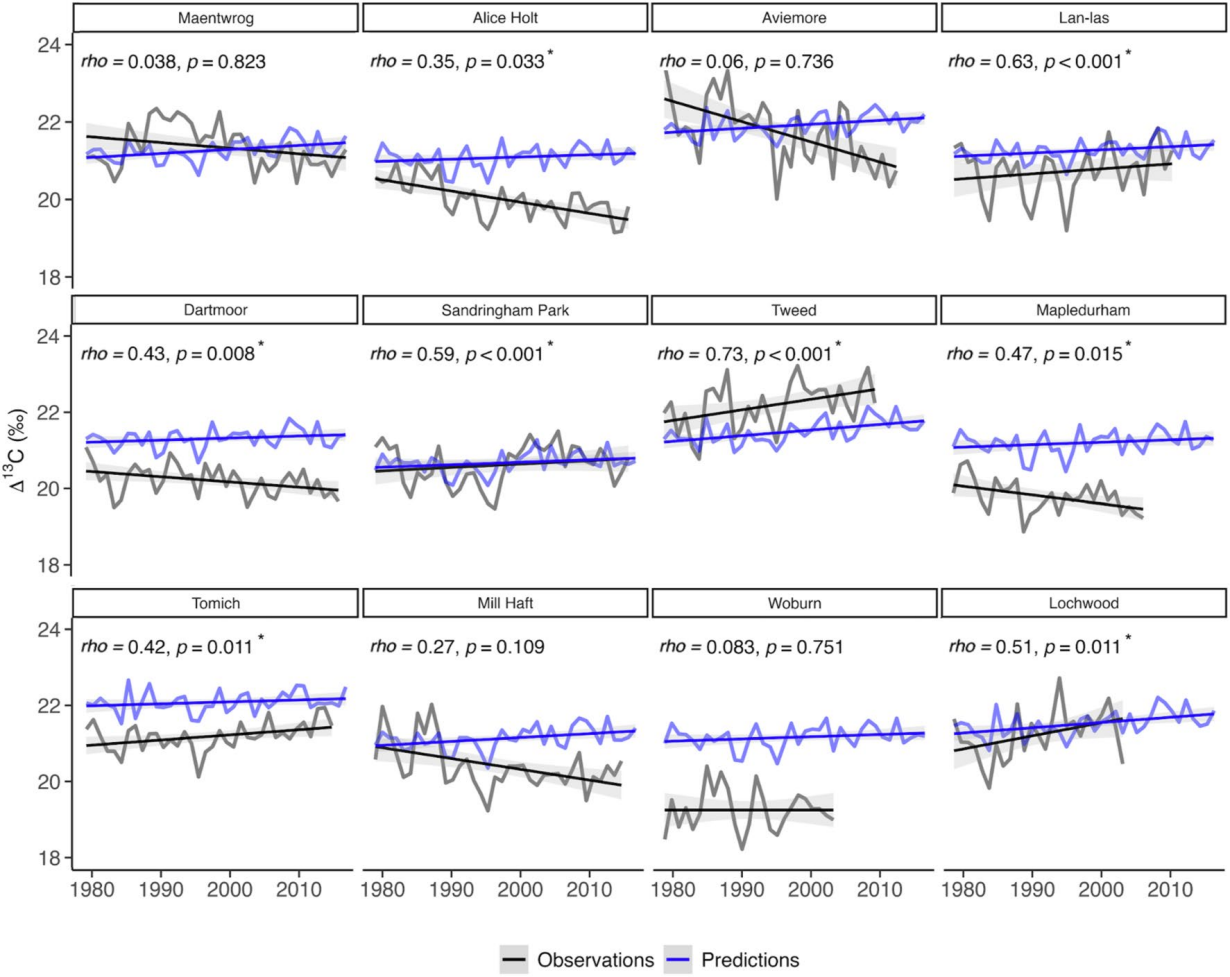
Measured (light blue) and modeled (dark blue) Δ^13^C at each site with respective trends over 1979–2016. The correlation coefficient (rho) indicates the strength of the correlation between the two variables, p denotes the probability value and * denotes statistical significance (*p* < 0.05).

Δ^13^C_TR_ and Δ^13^C_predicted_ were significantly correlated at eight out of the 12 sites Figure 2). The strongest relationships were found in Tweed (rho = 0.73, *p* < 0.001), Lan‐las (rho = 0.63, *p* < 0.001), Sandringham Park (rho = 0.59, *p* < 0.001), Lochwood (rho = 0.51, *p* < 0.05), Mapledurham (rho = 0.47, *p* < 0.05) and Dartmoor (rho = 0.43, *p* < 0.01), followed by Tomich (rho = 0.42, *p* < 0.05) and Alice Holt (rho = 0.35, *p* < 0.05). JULES overestimated Δ^13^C values in Alice Holt, Dartmoor, Tomich, Mill Haft, Lan‐las, Mapledurham and Woburn, but underestimated them in the Tweed catchment.

**Figure 3:**
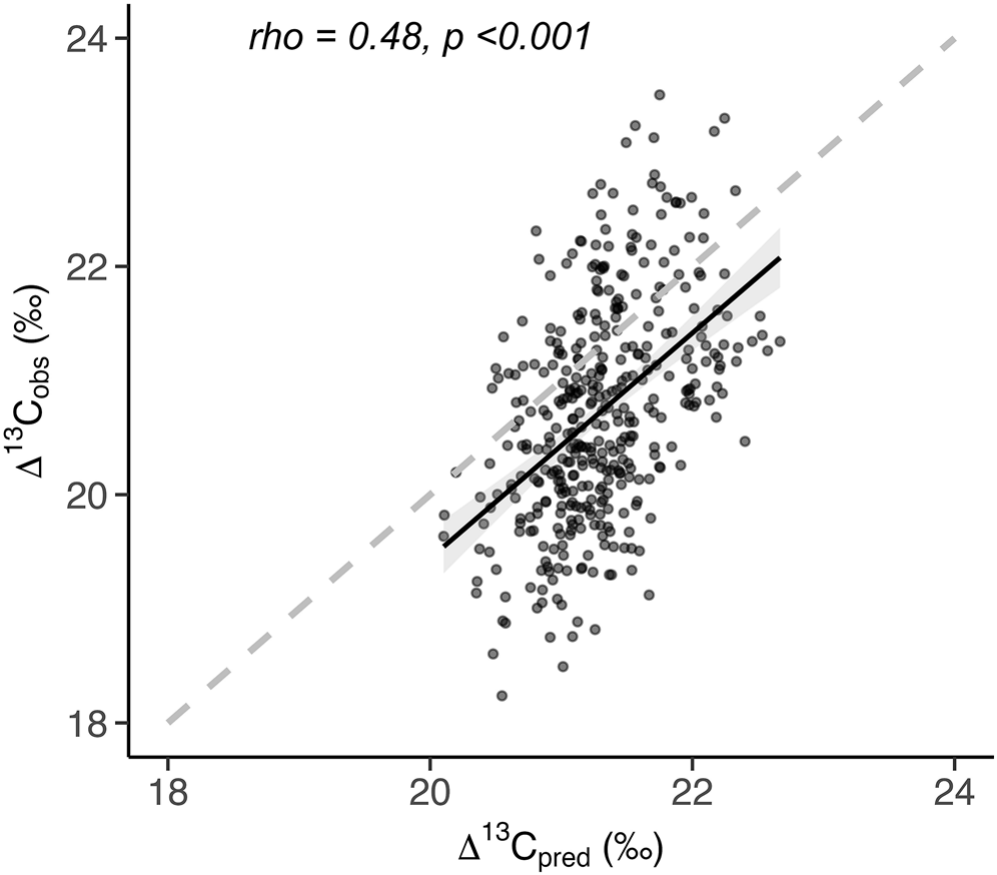
Δ^13^C_TR_ versus Δ^13^C_predicted_ (permil) across all sites and the 38‐year period, with a 1:1 line and spearman's rank correlation coefficient.

In addition to single‐site analysis, the tree‐ring derived and modeled Δ^13^C chronologies for each site were combined into a single composite chronology representing the UK as a whole. Δ^13^C_predicted_ and Δ^13^C_TR_ composite chronologies were significantly related to each other (rho = 0.48, *p* < 0.001) (Table S1 in Supporting Information S1 and Figure 3), indicating an overall high skill of JULES for predicting Δ^13^C in UK broadleaf deciduous trees.

**Figure 4:**
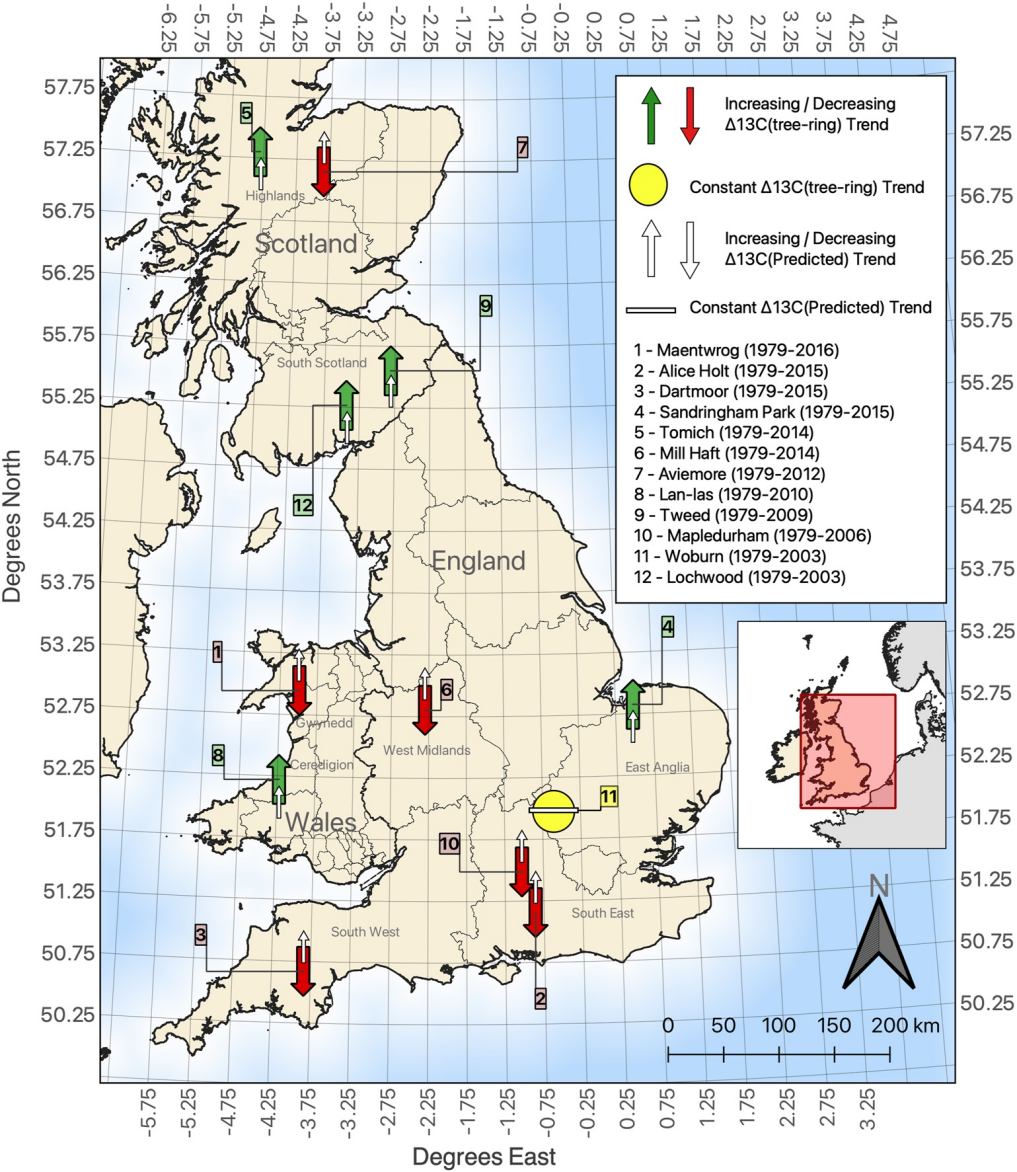
Predicted and tree‐ring based trends in Δ^13^C over the period 1979–2016 at each site.

While JULES simulated a rising or near‐constant trend in Δ^13^C at each site over the 38‐year period (Figure 2), tree‐ring derived Δ^13^C increased at five sites (Sandringham Park, Tomich, Lan‐las, Tweed and Lochwood), stayed constant at Woburn but decreased at the remaining six sites. Overall, Δ^13^C_TR_ tended to increase in Scotland but to decrease in southern England (Figure 4).

The inter‐annual variability of Δ^13^C was lower in the predictions than in the tree‐ring observations at all sites. While inter‐annual variability in Δ^13^C_predicted_ ranged from 0.25 in Dartmoor to 0.31 in Mapledurham and Woburn, that in Δ^13^C_TR_ varied from 0.37 in Tomich to 0.90 in Aviemore (Table 3). The largest differences in inter‐annual variability between predictions and observations were measured at Aviemore and Lan‐las. Conversely, the smallest disparities were depicted at Mapledurham and Tomich. Mean inter‐annual variations over the 12 sites were 49.29% lower in the predictions than in observations (Table 3). The Wilcoxon's test showed a statistically significant difference (*p* = 0.002) between measured and modeled Δ^13^C.

**Table 3:** Inter‐Annual Variability (Expressed as Standard Deviation of the Mean) of the Modeled and Measured Δ^13^C Chronologies at Each Site Over the Full 38‐Year Period

Site	Modeled variability	Measured variability
Maentwrog	0.28	0.57
Alice Holt	0.29	0.50
Dartmoor	0.25	0.39
Sandringham Park	0.27	0.53
Tomich	0.27	0.37
Mill Haft	0.30	0.64
Aviemore	0.30	0.90
Lan‐las	0.26	0.65
Tweed	0.30	0.62
Mapledurham	0.31	0.43
Woburn	0.31	0.55
Lochwood	0.29	0.65
Mean	0.29	0.57

**Figure 5:**
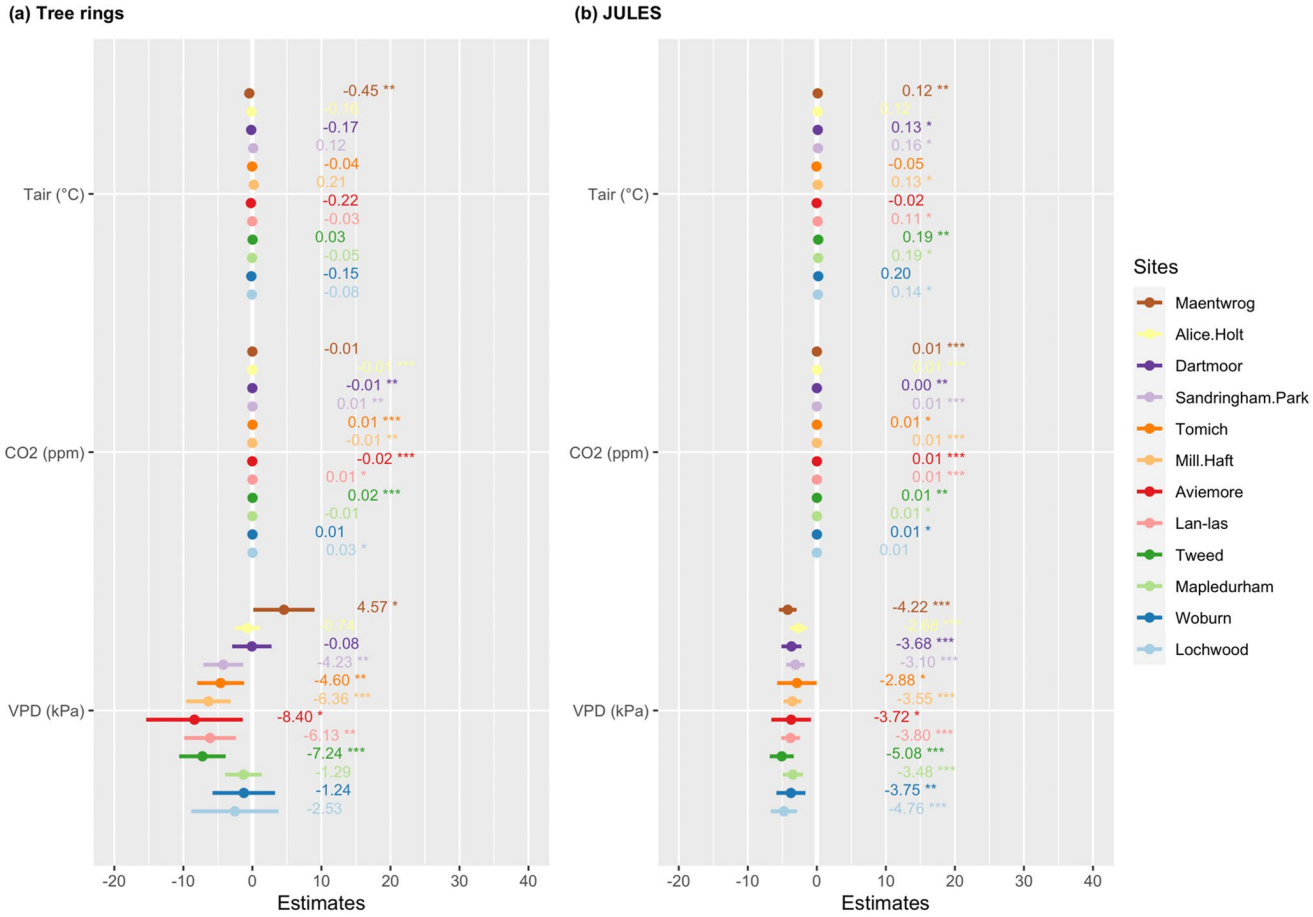
Comparison of the environmental controls (air temperature [*T*_air_], atmospheric carbon dioxide concentration and vapor pressure deficit) on Δ^13^C_TR_ and Δ^13^C_predicted_ at each site over the period 1979–2016, with regression coefficients. *: *p* < 0.05; **: *p* < 0.01; ***: *p* < 0.001.

The multiple regression models for individual sites included [CO_2_]_atm_, *T*_air_, and VPD, while the composite analysis also included the effect of elevation (*z*) on Δ^13^C. At some individual sites, the effect of *T*_air_ and VPD on Δ^13^C_TR_ was opposite to the overall response. Δ^13^C_TR_ increased significantly with [CO_2_]_atm_ at five sites and decreased at four. Δ^13^C_predicted_, however, increased with [CO_2_]_atm_ at 11 sites (Figure 5). Overall, the strongest environmental control on Δ^13^C_TR_ variability was from [CO_2_]_atm_ and VPD which were significant (*p* < 0.05) at nine and seven sites, respectively. While a rise in VPD decreased Δ^13^C_TR_ at six sites but increased it at one site, rising *T*_air_ significantly decreased Δ^13^C_TR_ at only one site. JULES predicted an increase in Δ^13^C with rising [CO_2_]_atm_ at 12 sites, an increase in Δ^13^C with rising *T*_air_ at eight sites, but a decrease in Δ^13^C with rising VPD at all sites of the network.

When considering all sites together, the environmental drivers from the multiple linear regression model explained 56% of variance in Δ^13^C_TR_ and 65% in Δ^13^C_predicted_ (Table 4). Overall, Δ^13^C_TR_ and Δ^13^C_predicted_ tended to decrease with both rising *T*_air_ and VPD. However, while Δ^13^C_TR_ stayed relatively constant with rising [CO_2_]_atm_ or increasing *z*, Δ^13^C_predicted_ increased with both [CO_2_]_atm_ and *z* (Table 4).

**Table 4:** Multiple Linear Regression Models of Δ^13^C_TR_ and Δ^13^C_predicted_ in All Sites Combined, Against Environmental Variables ([CO_2_]_atm_, *T*_air_, VPD, and Elevation [*z*]), Including Adjusted *R* ^2^, Root Mean Square Error (RMSE) and Regression Coefficients

Model		Overall model		Intercept	[CO_2_]_atm_ (ppm)	*T*_air_ (ºC)	VPD (kPa)	*z*(km)
	*n*	Adjusted *R* ^2^	RMSE	Coefficients				
Δ^13^C_TR_	396	0.56***	0.64	25.83***	ns	−0.26***	−1.48***	ns
Δ^13^C_predicted_	396	0.65***	0.27	20.51***	0.006***	−0.09***	−0.60***	2.27***

*Note*. ns: non‐significant; ***: *p* < 0.001

## 4 Discussion

The goal of the study was to evaluate the skills of JULES model at predicting inter‐annual variations of Δ^13^C in UK broadleaf oak trees and at capturing their response to local environmental conditions. Our results show that JULES was able to reproduce tree‐ring Δ^13^C variations at eight of the 12 study sites (Figure 2), with a maximum offset of 2.6‰ across all sites (Table 2). Inter‐annual variations in Δ^13^C_predicted_ were underestimated by up to 66.4% compared to the measured time series (Table 3). Additionally, while JULES predicted a near‐constant or rising trend in Δ^13^C, Δ^13^C_TR_ was more varying, increasing at five sites, decreasing at six sites, and staying constant at one site (Figures 2 and 4). The influences of *T*_air_ and VPD on Δ^13^C were reasonably well captured by the model, when considering all sites together (Table 4). When the individual sites were analyzed separately, the influence of [CO_2_]_atm_ and VPD on Δ^13^C was captured but overestimated at some sites. The model also failed in reproducing the directionality of some of the environmental impacts (Figure 5). In this section we discuss the reasons for (a) the offsets between measured and predicted Δ^13^C values, (b) the underestimation of the inter‐annual variability in Δ^13^C in JULES, (c) the weak agreement between measured and predicted Δ^13^C at four of the sites of the network, and (d) the spatio‐temporal variations in Δ^13^C across the UK.

### 4.1 Offsets Between Δ^13^C_TR_ and Δ^13^C_predicted_ Values Could Be Due To Biochemical Processes Not Incorporated in JULES

Churakova et al. (2015) found offsets of up to 4‰ between measured Δ^13^C values of tree ring cellulose and those predicted by ORCHIDEE model in Siberian larch trees. This was because the model did not account for post‐photosynthetic fractionations and consequent differences between the δ^13^C of photosynthates and cellulose. The offsets found here were lower than those from this study (2.6 vs. 4‰), because we accounted for these additional fractionations via the parameter *d* in the model (assuming *d* to be equal to 2.1‰ following average estimates from the literature; Frank et al., 2015). Lavergne et al. (2022) found that *d* varied across species and sites, but also across years, at least in an observational network from northern America (Guerrieri et al., 2016, 2019), and that the standard *d* value was likely underestimated. Their comparison of δ^13^C values between leaf and tree ring materials at the same sites rather suggested that *d* averages 4.1 ± 1.1‰. Thus, the residual offset of 2.6‰ found here could be due to underestimation of post‐photosynthetic fractionations at the study sites. Tcherkez et al. (2004) and Gessler et al. (2008) suggested that differential use of day‐ or night‐carbohydrates by leaf and stem material could influence the carbon isotopic signature of tree rings. Indeed, plant carbohydrates exported in the day are more depleted in ^13^C than those exported at night, causing isotopic partitioning between leaf and stem carbohydrates and differing Δ^13^C_TR_ mean values (Cernusak et al., 2009). Similarly, sucrose—the sugar transported from leaves for the formation of tree rings—is enriched in ^13^C relative to other water‐soluble carbohydrates in the leaf which may result in greater Δ^13^C_TR_ than Δ^13^C_predicted_ values as the leaf material is more depleted in ^13^C compared to the stem (Rinne et al., 2015). Further research is needed to better understand the factors controlling the post‐photosynthetic fractionations so that the value of *d* in JULES can be predicted at each site rather than being approximated as a single global estimate.

### 4.2 JULES Underestimates the Inter‐Annual Variability in Δ^13^C

The inter‐annual variability in Δ^13^C_predicted_ was underestimated compared to tree‐ring inferred Δ^13^C (Table 3). Thus, JULES failed to accurately simulate the variability in Δ^13^C over inter‐annual timescales. It is also possible that the coarse spatial resolution of the WFDEI‐WATCH forcing data set (0.5° × 0.5°) introduced disparities between site data and gridded input data which would increase model error, particularly regarding interannual variability in Δ^13^C. For instance, extracting July‐August Δ^13^C_leaf_ values from exact site‐specific grid points, may have contributed to increase the uncertainty in Δ^13^C_predicted_ values due to possible misrepresentation of local environmental conditions at some sites. Nevertheless, it is unlikely that this effect would have dampened the signal to the degree that is found here. Lavergne et al. (2022) also performed model evaluation against global Δ^13^C data using the WFDEI‐WATCH 0.5º × 0.5° forcing data set and found relatively good agreement between measurements and predictions (*r* = 0.54), with consistent underestimations of inter‐annual variability by JULES. We also tested an alternative approach, where modeled Δ^13^C values were extracted from the three closest grid points around each site and averaged (Figure S1 in Supporting Information S1). This approach, however, resulted in further dampening of the interannual variability signal in Δ^13^C_predicted_ resulting in greater disparities between JULES predictions and tree‐ring measurements (Table S2 in Supporting Information S1). The spatial agreement between Δ^13^C_predicted_ and Δ^13^C_TR_ was also decreased using this approach, with a significant correlation between Δ^13^C_predicted_ and Δ^13^C_TR_ at only one site (Figure S1 in Supporting Information S1) rather than eight when extracting Δ^13^C from specific coordinates (Figure 2).

Another explanation could be that the modeling approach used in JULES to predict leaf intercellular (*c*_i_) and chloroplastic (*c*_c_) partial pressures of trees to environmental changes needs to be improved. Lavergne et al. (2022) suggested that the Prentice model underestimates the interannual variability in measured Δ^13^C, because it does not consider the impact of soil moisture stress on stomatal activities. This is in line with a recent study showing that soil water limitation impacts leaf mesophyll conductance in Scots Pine (Leppä et al., 2022). Consistently, we found that interannual variability of Δ^13^C predicted by JULES was lower in the most drought‐stressed sites in the south and greater in the wetter northern sites (except Tomich). Finally, uncertainties in the tree‐ring measurements could also explain part of the discrepancies between Δ^13^C_predicted_ and Δ^13^C_TR._

### 4.3 The Agreement Between Δ^13^C_TR_ and Δ^13^C_predicted_ Is Weaker at Four Sites

Δ^13^C of structural carbohydrates in wood is closely linked to environmental change, therefore changes in environmental variables could have a greater impact on Δ^13^C_TR_ than on leaf‐level Δ^13^C (Diao et al., 2020; Loader et al., 2007). However, this is not consistent with Leppä et al. (2022) and Rinne et al. (2015) who both found a strong climate signal in leaf‐sugar δ^13^C variability. The weak correlations at the four sites may also be due to the soil water stress which was not accounted for by JULES. It is also possible that the impact of atmospheric pollution of sulfur dioxide (SO_2_) on plant material δ^13^C values may have dampened the environmental signal recorded in the tree rings. SO_2_ has been shown to induce stomatal closure (Linzon, 1972; Martin et al., 1988; Rinne et al., 2010; Savard et al., 2004) and consequently reduce Δ^13^C values. This effect is more prominent in broadleaved trees, such as *Quercus robur*, which are the dominant species considered in this study, than in *Pinus sylvestris* (Rinne et al., 2010). Despite a reduction of SO_2_ pollution due to modern air quality improvements, the effect of atmospheric pollution on stomatal functions should not be underestimated. Different concentrations of SO_2_ and other air pollutants at each site could indirectly influence the agreement between measured and predicted Δ^13^C values (Bodin et al., 2013; Rinne et al., 2010). It is possible that trees in Woburn (central England), for example, have been affected by atmospheric pollution from traffic and other industrial sources (Hemming et al., 1998). Despite a significant link between Δ^13^C_leaf_ and Δ^13^C_TR_ in Woburn, SO_2_ pollution could only explain part of the large offsets measured at this site (2.55‰). SO_2_ pollution is generally greater in England's industrial north and along the east coast where major power stations are located. Although the JULES simulations of Δ^13^C in and around these regions were significantly related to the tree‐ring observations (*p* < 0.05), both atmospheric SO_2_ and sulfur deposition may have contributed to disparities between Δ^13^C_TR_ and Δ^13^C_predicted_.

### 4.4 Spatio‐Temporal Variations in Δ^13^C Are Driven by Environmental Changes

Tree‐ring inferred Δ^13^C tended to increase in Scotland but to decrease in southern England, while JULES predicted an increase in Δ^13^C in both regions. This could be the imprint of different precipitation and temperature regimes in northern and southern UK and their influence on Δ^13^C. Mean annual rainfall in Scotland ranges between 600 and 3000 mm with mean annual temperatures of 4–9°C (Barnett et al., 2006; Werritty & Sugden, 2012). In contrast, mean annual temperature across the whole of England were around 10.6°C between 1981 and 2010 with mean annual precipitation of 989 mm (Kendon et al., 2021). Thus, in regions with relatively higher rainfall and lower temperature such as in Scotland, Δ^13^C increased because low *T*_air_ and VPD tend to have a positive effect on Δ^13^C.

PAR and plant‐available soil water can also impact Δ^13^C (Cernusak et al., 2013; Diefendorf et al., 2010; Hafner et al., 2014; Kohn, 2010; G. H. Young et al., 2010) but were not directly accounted for in the JULES simulations of Δ^13^C and thus excluded from our analyses. Terrestrial plants exhibit high Δ^13^C when photon flux density is low, however, the ratio of intercellular to ambient CO_2_ becomes independent of solar radiation above *c.* 250 μmol m^−2^ s^−1^ but there may still be a reduction in Δ^13^C because of transfer of leaf intercellular CO_2_ to CO_2_ concentration at RuBisCO (*c*_c_) (Cernusak et al., 2013; Ehleringer et al., 1986; Evans et al., 1986; Farquhar et al., 1989; Farquhar & Wong, 1984; Wong et al., 1978). Voelker et al. (2014) also note the potential negative correlation between irradiance and Δ^13^C, which is reflected in their results at the canopy level. It has been suggested that solar irradiance, rather than *T*_air_, controls tree‐ring carbon isotope composition in areas where moisture is not a limiting factor and where isotopic fractionation is controlled by the rate of photosynthesis (G. H. Young et al., 2010). Low soil water availability limits diffusion of CO_2_ through the stomata and results in lower values of Δ^13^C in tree rings (Cernusak et al., 2013). Diefendorf et al. (2010) and Kohn et al. (2010) also found ^13^C discrimination to decrease with decreases in mean annual precipitation. Accounting for the effects of these environmental variables in JULES could lead to more realistic Δ^13^C simulations across UK sites.

Keeling et al. (2017) suggested that Δ^13^C increased globally with rising CO_2_ concentration by 0.014 ± 0.007‰ ppm^−1^ over 1978–2014 due to both photorespiratory and mesophyll effects. Even though the increase in Δ^13^C predicted by JULES across the UK over 1979–2016 tends to agree with their findings, tree‐ring Δ^13^C time series suggest a decline rather than an increase in Δ^13^C over the same period at least in southern England. The discrepancies between their study and our may be partly related to the approach used by Keeling et al. (2017) who only considered the positive effect of CO_2_ on Δ^13^C but ignored the additional effects of *T*_air_ and VPD on Δ^13^C.

The composite chronologies of Δ^13^C from both the observational tree‐ring network and the model predictions suggest that *T*_air_ has a negative effect on Δ^13^C in the UK. This contrasts with Lavergne et al. (2022) who investigated the environmental drivers of Δ^13^C using the same JULES simulations at the global scale, and for different climatic regions. This relationship also differs from theoretical expectations (Cernusak et al., 2013). We would expect a negative effect of increasing VPD on Δ^13^C due to VPD‐induced stomatal closure, but a positive effect of rising *T*_air_ on Δ^13^C due to its negative impact on photorespiration (see Lavergne et al., 2022 and references therein). While the regression models at the individual sites showed a compelling negative VPD effect on Δ^13^C in both observations and predictions, the *T*_air_ effect on Δ^13^C was only significantly negative at one site (Maentwrog) for the tree‐ring network and significantly positive at eight sites in the model predictions. The negative *T*_air_ effect found in the composite analyses may therefore be an artefact and not representative of the physiological response of oak trees to *T*_air_ in the UK.

Δ^13^C tend to be lower at high altitudes where atmospheric pressure is relatively low, but higher at lower altitudes (Körner et al., 1988; Zhu et al., 2010). This elevation effect on Δ^13^C_TR_ was, however, not apparent at our sites. For instance, the two highest elevation sites in this study, that is, Aviemore (300 m) and Tweed (190 m) displayed the highest Δ^13^C values. Nevertheless, the range of elevation between study sites was relatively low (max. 250 m) suggesting that the elevation effect was not evident in our tree‐ring network.

Although our findings suggest that the overall modeled effect of climate on Δ^13^C in JULES is reasonably well predicted, the model needs to be improved to fully capture the response of Δ^13^C to individual environmental conditions. Adding more broadleaf deciduous sites in the UK tree‐ring network will help to increase understanding of spatio‐temporal variations in Δ^13^C in the UK and model‐data comparisons. In addition, the results of this study may be combined with further forest‐level measurements (e.g., tree‐ring width, local carbon flux data, or oxygen isotopes) to constrain modeled and measured stomatal activities to a greater extent (Raczka et al., 2016), thus improving predictions of tree response to future climate. In particular, the implementation of tree‐ring δ^18^O measurements has potential to lead to more accurate simulations of precipitation, temperature, relative humidity (Rinne et al., 2013), and therefore improved projections of future climate change.

## 5 Conclusions

The weak skills of models to predict stable carbon isotope variations is a widespread issue in LSM. Comparing the impacts of local environmental conditions on measured and modeled Δ^13^C is an effective approach to test models such as JULES and highlight areas for their improvements. We have shown here that JULES is able to reproduce long‐term variations in Δ^13^C as reconstructed by tree rings at eight of the 12 study sites but tend to underestimate the mean Δ^13^C values likely due to assumptions made around post‐photosynthetic fractionations. The inter‐annual variations in predicted Δ^13^C were underestimated in all sites of the network compared to the measured time series. Consistent with the literature, we found that *T*_air_ [CO_2_]_atm_ and VPD strongly influence variability in tree‐ring based Δ^13^C on interannual timescales. The environmental dependencies of tree‐ring Δ^13^C were relatively well captured by JULES but the model failed to capture the direction of change in Δ^13^C to *T*_air_, [CO_2_]_atm_, and VPD at some individual sites. Model refinements are required for improving predictions of mean, inter‐annual variations, and trends in Δ^13^C, and to reproduce local environmental influences. As the spatial resolution of measured carbon isotope values used this study is limited in extent, we recommend that a larger network of tree‐ring carbon isotopes is developed to improve understanding of spatio‐temporal trends in Δ^13^C for broadleaf deciduous trees across the UK.

## Supporting information

Supporting Information S1

## Data Availability

The carbon isotope data (tree‐ring derived Δ^13^C and JULES‐predicted Δ^13^C), environmental data and JULES output for each site as well as R script, full JULES simulations and WFDEI‐WATCH data used in this study are available at the following repository: https://zenodo.org/badge/latestdoi/487340507. The model code and the files needed for running it are available from the Met Office Science Repository Service (MOSRS; https://code.metoffice.gov.uk/trac/jules/; registration required). The results presented in this paper were obtained by running JULES vn5.6 branch with a new carbon isotopic modeling capability (code available after registration with MOSRS at https://code.metoffice.gov.uk/trac/jules/browser/main/branches/dev/alienorlavergne/vn5.6_jules_Cisotopes). The runs were performed with the Rose suite u‐bx886 (https://code.metoffice.gov.uk/trac/roses-u/browser/b/x/8/8/6).
