# Supplementary material for: Spatio‐Temporal Variations in Carbon Isotope Discrimination Predicted by the JULES Land Surface Model
Source: J Geophys Res Biogeosci. 2022 Dec 8;127(12):e2022JG007041. doi: 10.1029/2022JG007041 (PMC10078459; doi:10.1029/2022JG007041)
Supplement: Supplementary file 1 — Supporting Information S1 [file JGRG-127-0-s001.docx]

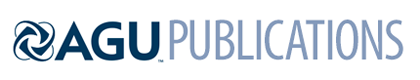


*Journal of Geophysical Research Biogeosciences*

Supporting Information for

Spatio-Temporal Variations in Carbon Isotope Discrimination Predicted by the JULES Land Surface Model

**Lewis Palmer ^a^, Iain Robertson ^a^, Aliénor Lavergne ^b, c^, Deborah Hemming ^d^, Neil Loader ^a^, Giles Young ^e^, Darren Davies ^a^, Katja Rinne-Garmston ^e^, Sietse Los ^a, f^, Jamie Williams ^a^**

**^a^ Department of Geography, Swansea University, Swansea, UK**

**^b^ Department of Geography and Environmental Science, University of Reading, Reading, UK**

**^c^ Department of Physics, Imperial College London, London, UK**

**^d^ Met Office, Exeter, UK**

**^e^ Natural Resources Institute Finland (Luke), Helsinki, Finland**

**^f^ Wetland Conservation Unit, Wildfowl and Wetland Trust (WWT), Gloucestershire, UK**

Corresponding author: Lewis Palmer ([lpalmer98@yahoo.co.uk](mailto:lpalmer98@yahoo.co.uk))

**Contents of this File:**

Table S1

Table S2

Figure S1

Supplementary:

**Table S1** – Spearman’s rank correlation coefficient between Δ^13^C_TR_ and Δ^13^C_predicted_ for all sites combined between 1979 and 2016.

| **Site** | **Spearman’s *rho*** | ***p*-value** | ***n*** | **RMSE** |
| --- | --- | --- | --- | --- |
|  |  |  |  |  |
| Composite of Sites | 0.48 | <0.001 | 396 | 1.02 |

**Table S2** – Inter-annual variability (expressed as standard deviation of the mean) of the modelled and measured Δ^13^C chronologies at each site, with over the full 38-year period. Model output was extracted from the three closest latitude/longitude grid points around each site and averaged.

| **Site** | **Measured Variability** | **Modelled**  **Variability** |
| --- | --- | --- |
| Maentwrog | 0.28 | 0.57 |
| Alice Holt | 0.29 | 0.50 |
| Dartmoor | 0.25 | 0.39 |
| Sandringham Park | 0.27 | 0.53 |
| Tomich | 0.27 | 0.37 |
| Mill Haft | 0.30 | 0.64 |
| Aviemore | 0.30 | 0.90 |
| Lan-las | 0.26 | 0.65 |
| Tweed | 0.30 | 0.62 |
| Mapledurham | 0.31 | 0.43 |
| Woburn | 0.31 | 0.55 |
| Lochwood | 0.29 | 0.65 |


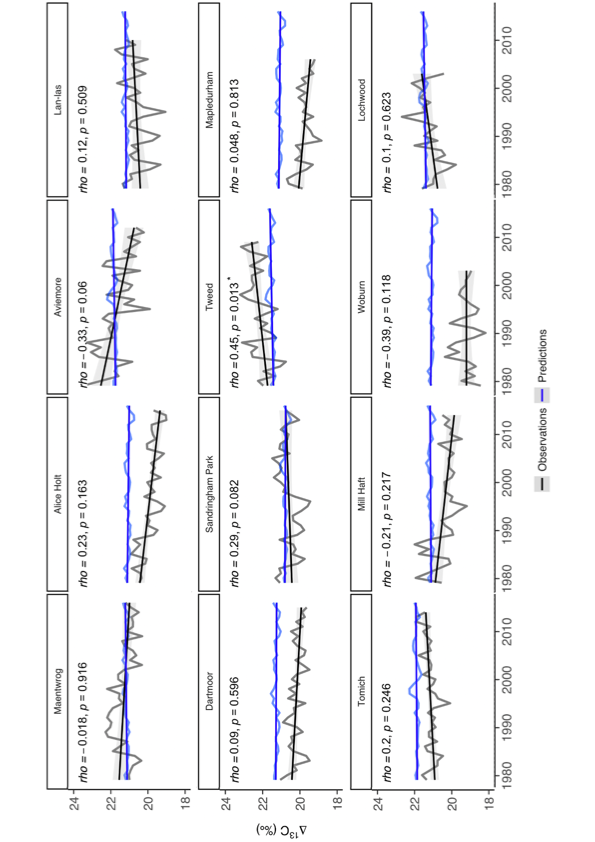


**Figure S1** - Measured (light blue) and modelled (dark blue) Δ^13^C at each site (extracted from the three closest latitude/longitude grid points around each site) with respective trends over 1979-2016. The correlation coefficient (rho) indicates the strength of the correlation between the two variables, p denotes the probability value, and an asterisk denotes a statistical significant relationship.
